# Mosquito transmission of the rodent malaria parasite *Plasmodium chabaudi*

**DOI:** 10.1186/1475-2875-11-407

**Published:** 2012-12-06

**Authors:** Philip J Spence, William Jarra, Prisca Lévy, Wiebke Nahrendorf, Jean Langhorne

**Affiliations:** 1 Division of Parasitology, MRC National Institute for Medical Research, Mill Hill, London NW7 1AA, UK

## Abstract

**Background:**

Serial blood passage of *Plasmodium* increases virulence, whilst mosquito transmission inherently regulates parasite virulence within the mammalian host. It is, therefore, imperative that all aspects of experimental malaria research are studied in the context of the complete *Plasmodium* life cycle.

**Methods:**

*Plasmodium chabaudi chabaudi* displays many characteristics associated with human *Plasmodium* infection of natural mosquito vectors and the mammalian host, and thus provides a unique opportunity to study the pathogenesis of malaria in a single infection setting. An optimized protocol that permits efficient and reproducible vector transmission of *P. c. chabaudi* via *Anopheles stephensi* was developed.

**Results and conclusions:**

This protocol was utilized for mosquito transmission of genetically distinct *P. c. chabaudi* isolates, highlighting differential parasite virulence within the mosquito vector and the spectrum of host susceptibility to infection initiated via the natural route, mosquito bite. An apposite experimental system in which to delineate the pathogenesis of malaria is described in detail.

## Background

*Plasmodium chabaudi chabaudi* establishes synchronous, chronic and recrudescing blood-stage infections in rodents, and exhibits many characteristics associated with the pathogenesis of human malaria, such as rosetting, sequestration and antigenic variation [1]. Genetically distinct isolates of *P. c. chabaudi* display distinct virulence phenotypes [2], and the development of *P. c. chabaudi* specific transfection technology has recently been reported [3]. *Plasmodium chabaudi' chabaudi* is therefore an ideal species with which to study the interaction between *Plasmodium* and the mammalian host in an *in vivo* setting. Nevertheless, it is established that serial blood passage of *Plasmodium* increases virulence [4,5], and it has been demonstrated that mosquito transmission of serially blood passaged *P. c. chabaudi* intrinsically modifies the asexual blood-stage parasite, transforming the course and outcome of infection [Spence PJ, Jarra W, Lévy P, Reid AJ, Chappell L, Brugat T, Sanders M, Berriman M, Langhorne J. *personal communication*. Mosquito transmission of *Plasmodium* therefore directly regulates parasite virulence, and is thus an imperative in experimental malaria research. The required conditions for transmission of *P. c. chabaudi* via the mosquito vector differ markedly from all other rodent malaria species [6,7]. An optimized protocol for mosquito transmission of *P. c. chabaudi* has been developed to facilitate efficient and reproducible transmission of distinct isolates of *P. c. chabaudi* to distinct strains of laboratory mice. Here, this protocol is presented, and details the requirements of transmission and the expected outcomes of infection. Mosquito transmission of *P. c. chabaudi* permits analysis of parasite virulence, and vector and host responses to infection, in the context of the complete *Plasmodium* life cycle. An apposite experimental system in which to study many of the hallmark features of human disease is described.

## Methods

### Mice, mosquitoes and parasites

Inbred BALB/c, C57BL/6 and C3H/HeN mice, and outbred Parkes mice, are bred under specific pathogen-free conditions at the MRC National Institute for Medical Research (NIMR). Experiments are performed in accordance with UK Home Office regulations (PPL 80/2358) and approved by the ethical review panel at the NIMR. Female C57BL/6 mice are routinely used to initiate mosquito transmission of *P. c. chabaudi* between six to eight weeks of age and at approximately 20 g body weight. Mice are housed under normal light conditions (light 07.00-19.00 : dark 19.00-07.00) at 22°C, 50% relative humidity and with continuous access to mouse breeder diet and water.

*Anopheles stephensi*, strain SD500, are reared at 28°C, 60% relative humidity and under normal light conditions (light 07.00-19.00 : dark 19.00-07.00). Egg lays are induced through live rat feeds and larvae are reared at a density of approximately 500 per litre of dH_2_O. Larvae are provided with Liquifry No. 1 and pool pellets, whilst adult mosquitoes are provided with Fructose. Female *An. stephensi* are routinely infected with *P. c. chabaudi* at seven to nine days of age. Following infection, mosquitoes are housed under normal light conditions at 26°C, 60% relative humidity and with continuous access to Fructose + PABA.

Frozen stocks of serially blood passaged *P. c. chabaudi* (isolates AS and CB) are maintained in liquid nitrogen, and can be obtained from the European Malaria Reagent Repository, University of Edinburgh. *Plasmodium chabaudi chabaudi* is routinely transmitted between C57BL/6 mice and *An. stephensi*. To initiate transmission, frozen parasite stocks are supplemented with a 0.5 volume of Krebs saline + glucose [3], rapidly thawed by hand and injected intraperitoneally (ip) into a single C57BL/6 mouse. Peripheral parasitaemia is monitored daily on thin blood smears and parasitized erythrocytes (pE) are isolated at a parasitaemia of 5-10%, typically day 5 post-injection, and used to infect mice.

## Materials

4-Aminobenzoic acid (PABA; Sigma, cat. no. A9878)

Atipamezole hydrochloride, 5mg/ml solution for injection (Atipam; veterinary prescription only)

Autoclavable mouse breeder diet, WO/HS (LabDiet, cat. no. 5021)

Cell culture dish, 60 x 15mm (Corning, cat. no. 430166)

Chromatography filter paper, 3MM (Fisher Scientific, cat. no. FB59515)

Collapsible cage, 30cm^3^ (BioQuip Products, Inc., cat. no. 1450B)

Compressed gas: CO_2_.

Cotton wool balls

Cover slips, glass, 18mm^2^ (Menzel Gläser, cat. no. MNJ-350-010 K)

D-(−)-Fructose (Sigma, cat. no. F0127)

Erlenmeyer conical flask, glass, 100ml (Fisher Scientific, cat. no. FB33131)

FBS Gold (PAA Laboratories, cat. no. A15-649)

Filter paper circles, grade 1, 125mm (Whatman, cat. no. 1001 125)

Gentamicin solution, 50mg/ml (Sigma, cat. no. G1397)

Glass basin, flat base, straight side, 300ml (VWR, cat. no. 216–2764)

Glucose (BDH (VWR), cat no. 101174Y)

Homogeniser mortar and pestle, 0.1ml (Fisher Scientific, cat. no. FB56673)

Ketamine hydrochloride, 100mg/ml solution for injection (Vetalar V; veterinary prescription only)

Liquifry No. 1 (Interpet)

Medetomidine hydrochloride, 1mg/ml solution for injection (Medetor; veterinary prescription only)

Microscope slides, ground edges 45° (VWR, cat. no. 631–1560)

Micro test tubes, 1.5ml (Eppendorf, cat. no. 0030 120.086)

Needle, 27G and 30G, 0.5 inch (BD, cat. no. 300635 and 304000)

PBS, pH 7.2 (Invitrogen, cat. no. 20012)

Polypropylene centrifuge tubes, 50ml (Corning, cat. no. 430291)

Pool pellets (Dr Clarke’s pond foods)

RPMI 1640 + L-Glutamine (Invitrogen, cat. no. 21875)

Rubber elastic bands

Sodium bicarbonate (Sigma, cat. no. S6297)

Sodium chloride solution, 0.9% (saline; Sigma, cat. no. S8776)

Syringe, 1.0ml (BD, cat. no. 300013)

Tulle fabric netting, white, 12cm^2^

Vacuum filter system, PES, 0.22μm pore size, 500ml (Corning, cat. no. 431097)

White premium paper cups, 12cl and 25cl (Benders, cat. no. 820400E and 8209006)

### Reagent set-up

Fructose: 8% (w/v) D-(−)-Fructose in dH_2_O. Sterilise by membrane filtration. Store at 4°C for up to one month.

Fructose + PABA (F/PABA): 8% (w/v) D-(−)-Fructose and 0.05% (w/v) PABA in dH_2_O. Sterilise by membrane filtration. Store at 4°C for up to one month and protect from light.

F/PABA + Gentamicin: 50μg/ml Gentamicin in F/PABA. Prepare a fresh solution before use.

RPMI + Glucose: 0.2% (w/v) sodium bicarbonate, 10% (v/v) FCS and 0.2% (w/v) glucose in RPMI 1640 + L-Glutamine. Prepare a fresh solution before use.

Ketamine + Medetomidine: 10mg/ml Ketamine hydrochloride and 100μg/ml Medetomidine hydrochloride in saline. Inject a 20g mouse with a 100μl volume to achieve the required working dose of 50mg/kg and 500μg/kg, respectively. Prepare a fresh solution before use.

Atipamezole: 1mg/ml Atipamezole hydrochloride in saline. Inject a 20g mouse with a 50μl volume to achieve the required working dose of 2.5mg/kg. Prepare a fresh solution before use.

### Equipment

Improved Neubauer cell counting chamber, depth 0.1mm (Hawksley, cat. no. AC1000)

Insect aspirator, 1.5V powered (Hausherr’s machine works)

Light microscope, phase contrast, 10 - 40x objectives (Zeiss Axio Scope A1 or equivalent)

Microcentrifuge (Heraeus Fresco 17 or equivalent)

Stereomicroscope, 7.5 - 60x zoom (Leica M80 or equivalent)

Ultrasonic humidity cabinet (LEEC SFC3C/RH or equivalent)

### Procedure

Day −**14** | C57BL/6 mice are injected ip with 10^5^ pE and peripheral parasitaemia is monitored by thin blood smear throughout infection. For every 100 mosquitoes that are to be infected ≥1 mouse with a gametocyte density ≥0.1% of total erythrocytes will be required; the number of mice initially infected is, therefore, dependent upon the frequency that will exceed that threshold. Note: Transmission fails if circulating gametocytes are not observed, and transmission efficiency is substantially reduced if gametocytes are observed at a density <0.1% of total erythrocytes. Critical: Some isolates of *P. c. chabaudi* are highly virulent, causing severe disease and mortality, and necessitating increased numbers and careful monitoring of mice.

Day −**5** | Up to 1,500 female *An. stephensi* (at two to four days of age) are transferred to a 30 cm^3^ collapsible cage, whose base is lined with three sheets of chromatography filter paper. A feeding station (a 100 ml conical flask with a chromatography filter paper wick and cotton wool bung) is filled with 50 ml of F/PABA + Gentamicin and placed within the cage. The cage is transferred to an ultrasonic humidity cabinet and mosquitoes are hereafter maintained continuously at 26°C, 60% relative humidity. Note: For every mouse that is to be infected 30 mosquitoes are initially transferred to the cage. Although only 20 mosquitoes are ultimately required per mouse, it is necessary to compensate for the attrition of mosquito numbers that will occur over the following 20 days.

Day −**2** | The feeding station is replaced to provide 50 ml of F/PABA.

Day −**1** | The feeding station is removed and sterile dH_2_O is provided by saturating cotton wool balls, placing them into the caps of 50 ml centrifuge tubes and placing these cotton wool caps onto the top of the cage; one cotton wool cap is provided for every 100 mosquitoes. Note: It is important to starve mosquitoes 24 hours prior to transmission.

Day **0** | A thin blood smear is prepared from all mice at approximately 14.00 hours; slides are fixed with methanol and stained with Giemsa’s working solution [3]. Gametocytes are enumerated in a count of 5,000 erythrocytes, and all mice that equal or surpass the gametocyte density threshold of 0.1% of total erythrocytes are selected for transmission. Selected mice are anaesthetized by ip injection of Ketamine + Medetomidine and placed ventral side down onto the top of the cage; mosquitoes are allowed to feed for 30 minutes at room temperature. Following transmission, sterile dH_2_O is provided to mosquitoes in cotton wool caps placed on the top of the cage, which is then returned to the ultrasonic humidity cabinet. Mice are culled by an approved method. NOTE: Transmission is performed at 18.00 hours and under low light conditions. CRITICAL: The required temperature for *P. c. chabaudi* fertilization and development within the mosquito vector is 26.0°C (+/− 0.5°C).

Day **1** | F/PABA is provided to mosquitoes in cotton wool caps placed on the top of the cage.

Day **2** | The cotton wool caps are replaced to provide fresh F/PABA.

Day **4** | The cotton wool caps are removed and a feeding station containing 50 ml of F/PABA is placed within the cage. An egg bowl is similarly placed within the cage, allowing mosquitoes to safely deposit their eggs. To prepare the egg bowl, a 300 ml glass basin is filled to the rim with dH_2_O, and a 125 mm filter paper circle (with a 15 mm hole cut from the centre) is positioned such that the outer edge of the circle adheres to the rim of the basin and the inner edge of the circle lies under the surface of the water.

Day **6** | The egg bowl is removed, and the feeding station is replaced to provide a further 50 ml of F/PABA.

Day **8** | To visualize oocyst development: 20 mosquitoes are harvested from the cage using an insect aspirator, anaesthetized with CO_2_ and transferred to a cell culture dish on ice; mosquito midguts are dissected under a stereomicroscope, transferred into PBS on a microscope slide and covered with a glass cover slip; midguts are observed under a light microscope, utilizing phase contrast to visualize and enumerate oocysts. The feeding station is replaced to provide remaining mosquitoes with a further 50 ml of F/PABA. Note: For guidance on the dissection of *Plasmodium*-infected mosquitoes refer to ‘Methods in *Anopheles* Research’ [8].

Day **10** | The feeding station is replaced to provide a further 50 ml of F/PABA.

Day **12** | The feeding station is replaced to provide a further 50 ml of F/PABA.

Day **14** | For every mouse that is to be infected, 20 mosquitoes are harvested from the cage using an insect aspirator, anaesthetized with CO_2_ and transferred to a 25 cl paper cup, which is covered with tulle fabric netting and secured with rubber elastic bands. Alternatively, 10 mosquitoes can be transferred to 25 cl paper cups, or single mosquitoes to 12 cl paper cups, to reduce the number of mosquito bites. An additional mosquito cup is prepared for enumerating sporozoites, and dH_2_O is provided to mosquitoes in cotton wool caps placed on the top of the paper cups. Note: It is important to starve the mosquitoes 24 hours prior to transmission. Critical: Mosquitoes are transferred to paper cups 24 hours prior to transmission to provide sufficient recovery time following anaesthesia. Transfer of mosquitoes to paper cups is not required if experimental mice are to be infected via mechanical transmission.

Day **15** | To enumerate sporozoites: 20 mosquitoes are anaesthetized with CO_2_ and transferred to a cell culture dish on ice; mosquito salivary glands are dissected under a stereomicroscope, transferred into 100 μl of RPMI + Glucose in a 0.1 ml homogenizer and maintained on ice; all sets of salivary glands are pooled and gently homogenized for 30 seconds to release sporozoites, and the supernatant is transferred to a 1.5 ml micro test tube; the sample is centrifuged at 3,500 x*g* in a microcentrifuge for 3 minutes at 4°C and washed 2x with 500 μl RPMI + Glucose; sporozoites are resuspended in 10 μl RPMI + Glucose, transferred to a cell counting chamber and enumerated under a light microscope utilizing phase contrast.

For natural transmission of *P. c. chabaudi* via mosquito bite, each mouse is anaesthetized by ip injection of Ketamine + Medetomidine and placed ventral side down onto the top of one mosquito cup; mosquitoes are allowed to feed for 20 minutes at room temperature. Mice are then injected ip with Atipamezole and monitored to recovery. The number of fed mosquitoes in every cup is enumerated immediately by anaesthetizing mosquitoes with CO_2_, or at a later time by storing the mosquito cups at −20°C. Note: Transmission is performed at 18.00 hours and under low light conditions. Critical: Mice must be closely monitored for recovery from anaesthesia; for example, by testing their righting reflex.

For mechanical transmission of *P. c. chabaudi* via injection, mosquito salivary glands are dissected and homogenized (as described above) and sporozoites washed 3x with 1 ml RPMI + Glucose. Sporozoites are resuspended in 100 μl RPMI + Glucose, enumerated on a cell counting chamber using 10 μl of sample, further diluted in RPMI + Glucose to the required concentration, and injected intravenously (iv) or intradermally (id) into mice using 27G or 30G needles, respectively. Note: To obtain sufficient sporozoite numbers, assume that 100 mosquitoes will yield approximately 25,000 (+/− 10,000) sporozoites. Critical: Dissected salivary glands and isolated sporozoites should be maintained throughout on ice, and the time from dissection to injection should be minimized to maximize sporozoite viability.

## Results

**Figure 1:**
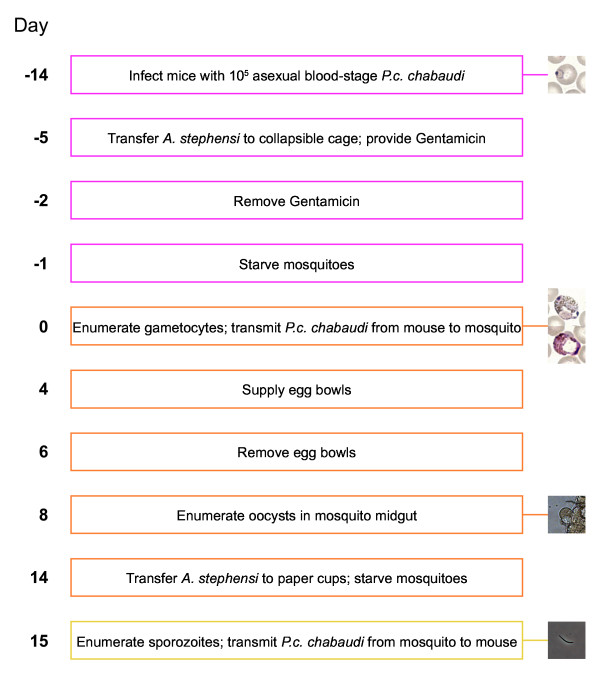
**Timeline of mosquito transmission of *Plasmodium chabaudi chabaudi *.** Prior to infection, *Anopheles stephensi* have continuous access to F/PABA from day −5 to day −1 and, following infection, from day 1 to day 14; F/PABA is replaced at ≤48-hour intervals. Images are shown for reference: *P. c. chabaudi* AS ring (day −14) and macro- (top) and micro- (bottom) gametocyte (day 0) infected erythrocytes isolated from wild-type C57BL/6 mice, stained with Giemsa’s working solution and visualized with light microscopy; *P. c. chabaudi* AS oocyst (day 8) and sporozoite (day 15) isolated from *An. stephensi* and visualized with phase contrast microscopy.

*Plasmodium chabaudi chabaudi* (isolates AS and CB) is routinely transmitted between wild-type C57BL/6 mice and *An. stephensi* utilizing this protocol (Figure 1). All available data from mosquito transmission of *P. c. chabaudi* over a one-year period (18 independent experiments) has been collated, and the results described herein derive from that pooled dataset, unless stated otherwise.

**Figure 2:**
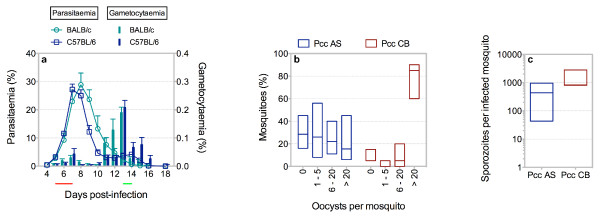
**Gametocytosis and sporogony in *Plasmodium chabaudi chabaudi*.a**, Parasitaemia and gametocytaemia of BALB/c and C57BL/6 mice infected with *P. c. chabaudi* AS by ip injection of 10^5^ pE (n = six per group; data are presented as mean with SEM). Transmission efficiency to *Anopheles stephensi* is highlighted under the graph: low efficiency (red line) *versus* high efficiency (green line). **b**-**c**, Number of oocysts in the midguts of *An. stephensi* on day 8 (**b**) and sporozoites isolated from the salivary glands of infected *An. stephensi* on day 14 (**c**) post-infection with *P. c. chabaudi* AS (Pcc AS) or *P. c. chabaudi* CB (Pcc CB) (n = 820; data are presented as median with range). The number of sporozoites in the salivary glands of *An. stephensi* infected with Pcc CB is significantly increased, as compared to those infected with Pcc AS (^*^p = 0.0222 (Mann–Whitney test)). C57BL/6 mice with a gametocyte density of ≥0.1% of total erythrocytes were utilized on day 14 post-infection at a ratio of ≥1 mouse to 100 mosquitoes.

Infection of C57BL/6 mice with serially blood passaged *P. c. chabaudi* AS via ip injection of pE leads to an early peak of circulating gametocytes, concurrent with the peak of asexual blood-stage parasitaemia, and a late peak of circulating gametocytes of increased magnitude that follows peak parasitaemia (Figure 2a). A similar kinetic of gametocytosis is observed in BALB/c mice, another widely used inbred laboratory strain. Transmission of *P. c. chabaudi* AS to *An. stephensi* early in infection (days 5, 6 or 7) is inefficient, whereas transmission late in infection (days 13 or 14) is highly efficient (data not shown). Importantly, *An. stephensi* must feed only on mice with a circulating gametocyte density ≥0.1% of total erythrocytes (at a ratio of ≥1 mouse to 100 mosquitoes) to maximize efficiency of transmission; of 526 C57BL/6 mice infected with *P. c. chabaudi* AS, 408 mice (77.6%) were equal to or surpassed this threshold on day 14 post-infection. Under these conditions, transmission of *P. c. chabaudi* AS results in infection of 71.5% (55% - 85%) (median with range) of mosquitoes; of 311 *An. stephensi* infected with *P. c. chabaudi* AS, 227 (73%) harboured between 1-20 oocysts on day 8 post-infection (Figure 2b). On day 14 post-infection, the number of salivary gland sporozoites in *An. stephensi* infected with *P. c. chabaudi* AS is 437.5 (43.2-956) (median with range) (Figure 2c). Interestingly, *P. c. chabaudi* CB, an isolate with increased virulence within the mammalian host, displays increased transmissibility, and *An. stephensi* infected with *P. c. chabaudi* CB harbour increased oocyst and sporozoite burdens (Figure 2b-c).

**Figure 3:**
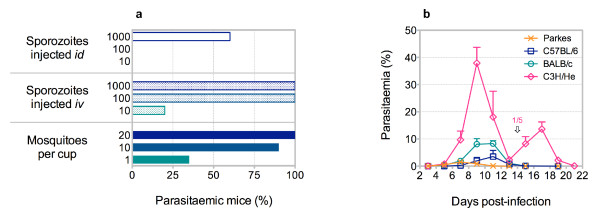
**Transmissibility and virulence of *Plasmodium chabaudi chabaudi *AS in the mammalian host. a,** Frequency of patent blood-stage parasitaemia in C57BL/6 mice injected id or iv with *P. c. chabaudi* AS sporozoites, or exposed to cups of *P. c. chabaudi* AS infected mosquitoes (n = 5-34 per group; pooled data from two independent experiments are shown). Parasitaemic mice were defined by thin blood smear, prepared daily between days 3 and 16 post-infection, with a limit of detection of 0.01% pE. **b**, Parasitaemia of Parkes, C57BL/6, BALB/c and C3H/He mice infected with *P. c. chabaudi* AS via mosquito bite (n = five per group; data are presented as mean with SEM). Peak parasitaemia is significantly increased in BALB/c mice, as compared to Parkes mice, and in C3H/He mice, as compared to BALB/c mice (^**^p = 0.0079 (Mann–Whitney test)). Note that on day 14 post-infection one C3H/He mouse succumbed to infection.

Sporozoites obtained from salivary glands of *An. stephensi* on day 15 of infection with *P. c. chabaudi* AS are highly infective; iv injection of 10 sporozoites is sufficient to elicit patent blood-stage parasitaemia in 20% of C57BL/6 mice, whilst 100 sporozoites ensures patent parasitaemia in 100% of mice (Figure 3a). Inoculation of sporozoites by id injection is comparatively ineffectual; 1,000 sporozoites results in just 60% of mice with patent parasitaemia. In contrast, transmission of *P. c. chabaudi* AS via mosquito bite is highly efficient; one third of all C57BL/6 mice exposed to single mosquito bites develop patent blood-stage parasitaemia, whilst exposure to 20 mosquitoes ensures patent parasitaemia in 100% of mice (Figure 3a). Although the course of infection in mice infected via mosquito bite is similar to that observed in mice injected with sporozoites, the potential influence of the route of transmission on the pathogenesis of malaria is not yet fully understood. Mice are therefore routinely infected with *P. c. chabaudi* via the natural route, and exposed to 20 *An. stephensi* leading to an estimated 9.15 (6.9-13.6) (median with range) infective bites; of 117 wild-type C57BL/6 mice infected with *P. c. chabaudi* AS via mosquito bite, 116 mice (99.1%) developed patent blood-stage parasitaemia. The pre-patent period in C57BL/6 mice infected with *P. c. chabaudi* AS via mosquito bite is 1 (<1-6) (median with range) day(s) following liver merozoite egress; 39 of 45 mice (86.7%) have a pre-patent period of ≤2 days. To observe a pre-patent period of ≤2 days in C57BL/6 mice infected with *P. c. chabaudi* AS via direct blood challenge would require iv injection of ≥10^5^ pE, approximating the number of liver merozoites that initiate the erythrocytic cycle following mosquito transmission. The resulting course of infection, and associated morbidity and mortality, within the mammalian host is strain dependent (Figure 3b); similarly, the course and outcome of infection is dependent upon the isolate of *P. c. chabaudi* inoculated via mosquito bite [Spence PJ *et al*. *personal communication*.]. Mosquito transmission of *P. c. chabaudi* therefore permits analysis of parasite virulence and host susceptibility to malaria, in the context of the complete *Plasmodium* life cycle and with infections initiated via the natural route.

## Discussion and conclusions

Mosquito transmission of *P. c. chabaudi* according to this optimized protocol is highly efficient and reproducible, and facilitates routine transmission of distinct isolates of *P. c. chabaudi* with a success rate of 100%. Optimal transmission is attained when *An. stephensi* feed on mice after the peak of asexual blood-stage parasitaemia, when circulating gametocyte densities surpass 0.1% of total erythrocytes. Thereafter, it is critical that infected mosquitoes are continuously maintained at 26.0°C; unfed mosquitoes are therefore not removed from cages, and mosquito handling is minimized at all times. This inevitably leads to an apparent limitation of the protocol; the number of infected mosquito bites is not known for individual mice. Nevertheless, the process of sporozoite inoculation in the mammalian dermis during mosquito probing is stochastic [9,10]; the sporozoite inoculum in all individuals, including those exposed to a defined number of infected mosquito bites, will always be unknown. Importantly, the inoculum size is unlikely to be associated with disease severity or outcome [11-13]. Indeed, it was noted in this study that the magnitude of peak parasitaemia is not influenced by the number of injected sporozoites, or the number of mosquito bites, that initiate infection. Thus, whilst in exceptional experimental circumstances it is necessary to control the pathogen dose via mechanical transmission of sporozoites [14], the majority of experimental studies of malaria should be initiated via mosquito bite, the natural route of sporozoite inoculation. In this context, it is of considerable importance that mosquito vector densities of *P. c. chabaudi*, unlike other rodent malaria species, are within the accepted physiological range associated with human *Plasmodium* infections of their natural vectors [15]. Mosquito transmission of *P. c. chabaudi* therefore provides an unparalleled opportunity to study both the mosquito vector and mammalian host stages of malaria within a single relevant infection setting. This protocol has been utilized to demonstrate that the vector directly regulates *Plasmodium* virulence during the erythrocytic cycle [Spence PJ *et al*. *personal communication*, and the consequence of vector regulation can clearly be observed when comparing the course of an infection initiated via mosquito bite, as opposed to following serial blood passage (Figure 2a and Figure 3b). It is therefore critical to perform experimental malaria studies, including those focused only on the erythrocytic cycle, in the context of the complete *Plasmodium* life cycle. Thus, mosquito transmission of *Plasmodium* should no longer be the exception but, rather, the rule in the field of experimental malaria.

## Competing interests

The authors declare that they have no competing interests.

## Authors’ contributions

PJS, WJ and JL designed the study; PJS and WJ developed the protocol; PJS, WJ, PL and WN performed the experiments; PJS analysed the data; PJS wrote the manuscript; all authors read and approved the final manuscript.
